# Relationships between the ABC-Exporter HetC and Peptides that Regulate the Spatiotemporal Pattern of Heterocyst Distribution in *Anabaena*

**DOI:** 10.1371/journal.pone.0104571

**Published:** 2014-08-14

**Authors:** Laura Corrales-Guerrero, Enrique Flores, Antonia Herrero

**Affiliations:** Instituto de Bioquímica Vegetal y Fotosíntesis, Consejo Superior de Investigaciones Científicas and Universidad de Sevilla, Seville, Spain; University of Groningen, Groningen Institute for Biomolecular Sciences and Biotechnology, Netherlands

## Abstract

In the model cyanobacterium *Anabaena* sp. PCC 7120, cells called heterocysts that are specialized in the fixation of atmospheric nitrogen differentiate from vegetative cells of the filament in the absence of combined nitrogen. Heterocysts follow a specific distribution pattern along the filament, and a number of regulators have been identified that influence the heterocyst pattern. PatS and HetN, expressed in the differentiating cells, inhibit the differentiation of neighboring cells. At least PatS appears to be processed and transferred from cell to cell. HetC is similar to ABC exporters and is required for differentiation. We present an epistasis analysis of these regulatory genes and of genes, *hetP* and *asr2819*, successively downstream from *hetC*, and we have studied the localization of HetC and HetP by use of GFP fusions. Inactivation of *patS*, but not of *hetN*, allowed differentiation to proceed in a *hetC* background, whereas inactivation of *hetC* in *patS* or *patS hetN* backgrounds decreased the frequency of contiguous proheterocysts. A HetC-GFP protein is localized to the heterocysts and especially near their cell poles, and a putative HetC peptidase domain was required for heterocyst differentiation but not for HetC-GFP localization. *hetP* is also required for heterocyst differentiation. A HetP-GFP protein localized mostly near the heterocyst poles. ORF *asr2819*, which we denote *patC*, encodes an 84-residue peptide and is induced upon nitrogen step-down. Inactivation of *patC* led to a late spreading of the heterocyst pattern. Whereas HetC and HetP appear to have linked functions that allow heterocyst differentiation to progress, PatC may have a role in selecting sites of differentiation, suggesting that these closely positioned genes may be functionally related.

## Introduction

In response to deprivation of combined nitrogen, some filamentous cyanobacteria produce cells called heterocysts that are specialized in the fixation of N_2_ [1]. Heterocyst differentiation involves drastic changes in gene expression that are coordinated by two DNA-binding factors, the global regulator NtcA and the development-specific factor HetR [2]. The distribution of heterocysts in the diazotrophic filaments of cyanobacteria represents a simple and old example of developmental patterns in the living world. In strains of the genera *Anabaena* and *Nostoc* the pattern consists of long linear chains of cells with heterocysts separated by ca. 10 vegetative cells. Several gene products that influence the pattern of heterocyst distribution have been identified [2]. In *Anabaena* sp. strain PCC 7120 (hereafter *Anabaena*) the *patS* gene is expressed early in the differentiation process, specifically in the differentiating cells, and inhibits the differentiation of neighboring cells [3], [4]. Inactivation of *patS* produces a Mch (Multiple contiguous heterocysts) phenotype whereas overexpression of *patS* abolishes differentiation. The primary product of *patS* is a 17-amino acid peptide [5]. The 9-amino acid N-terminal stretch of PatS appears to be involved in processing the peptide, which is needed for immunity against PatS in the differentiating cells in which the peptide is produced. Processing of PatS would render a C-terminal peptide, likely of 8 amino acids, that acting as a morphogen is transferred to the neighboring vegetative cells [5]. PatS appears to interact with HetR and regulate its activity [6], [7], [8], but the pathway of intercellular transfer of PatS or a peptide derivative of PatS is unknown.

The *hetN* gene product, which exhibits similarity to short chain dehydrogenases, also affects the pattern of distribution of heterocysts in the filament [9], [10]. *hetN* is expressed as a monocistronic transcript starting ca. 6–12 h after N (nitrogen) step-down [10]. Contrasting results have been reported when *hetN* was inactivated by insertion of different constructs (or when *hetN* was over-expressed). A Δ*hetN* strain has recently been reported to yield increased heterocyst frequency and Mch 48 h after N step-down [11]. Finally, inactivation of *patS* together with under-expression of *hetN* produced massive heterocyst differentiation in the filaments of *Anabaena* [12]. Aside from the speculation that a RGSGR peptide resulting from hydrolysis of HetN in the cytoplasm would likely be transferred to neighboring cells directly through inter-cytoplasmic connections [11], neither the identity nor the mechanism of action of the actual HetN-derived signaling molecule is known.

The *hetC* gene product exhibits extensive similarity to ABC transporters, especially to those in the HlyB family of bacterial protein exporters [13], [14]. *hetC* is induced early during heterocyst differentiation and is regulated by NtcA and HetR [13], [15], and certain of its mutants do not form heterocysts [13]. However, after prolonged incubation in the absence of combined nitrogen, *hetC* mutants exhibit a pattern of weakly-fluorescent cells, a characteristic of heterocysts [13], but, in contrast to heterocysts, they can divide producing a pattern of spaced series of small cells [16]. Because heterocysts are terminal, non-dividing cells, this observation led to the proposal that HetC is involved in the transition to non-dividing cells during heterocyst differentiation [16].

Genes *hetP* and *asr2819* are located downstream from *hetC* in the genome of *Anabaena*. The product of *hetP* bears no similarity to proteins of known function, and inactivation of *hetP* blocks heterocyst differentiation whereas its over-expression from a plasmid leads to over-differentiation [17], [18]. Because ectopic expression of *hetP* (from P*_petE_* in a plasmid) in a *hetR* mutant leads to the formation of heterocyst-like cells, which however do not fix N_2_ aerobically, it has been proposed that HetP partially bypasses the requirement for HetR acting downstream from it during heterocyst differentiation [18]. Also, because in a Δ*hetP* mutant the pattern of expression of *gfp* transcriptional fusions to *patS* or *hetR* appeared similar to those in the wild type background, *hetP* was suggested to function downstream of pattern formation during heterocyst differentiation [18].

In this work, we have addressed the relationships of HetC with possible partners regulating heterocyst differentiation. We have investigated whether HetC may be involved in the export of portions of PatS and HetN from differentiating cells and whether the predicted peptidase domain of HetC may be involved in processing of PatS. Additionally, we have investigated possible relationships of HetC with the products of the heterocyst differentiation gene *hetP* and the previously unstudied gene *asr2819*, which are genomic neighbors of *hetC*. The study has also included analyses of the subcellular localization of some of these factors.

## Materials and Methods

### Strains and growth conditions

*Anabaena* sp. PCC 7120 and derivatives were grown photoautotrophically in a BG11_0_-based medium supplemented with NH_4_Cl as described [19]. For bubbled cultures, the medium was supplemented with NaHCO_3_ and sparged with a mixture of air/CO_2_ (99∶1). Antibiotics, when required, were added to the media at final concentrations of: neomycin (Nm) 10 µg·mL^−1^, and each of streptomycin and spectinomycin 2 µg·mL^−1^ for liquid and 5 µg·mL^−1^ for solid cultures. For growth tests in solid medium, filaments grown in liquid BG11_0_ medium supplemented with NH_4_^+^ were washed 3 times with BG11_0_. Amounts of filaments corresponding to 5, 2.5, 1.25, 0.625 and 0.3125 ng Chl were spotted atop Petri dishes containing BG11_0_ medium supplemented or not with NH_4_^+^, without antibiotics, and the plates were incubated under standard conditions for 14 days.

### Construction of mutants

To replace the *hetC* gene (*alr2817*) with the Sm^r^ Sp^r^ gene cassette C.S3, which includes transcriptional terminators at both ends [20], two DNA fragments, one encompassing sequences 5′ of the gene and the other including sequences 3′ of the gene, were amplified by PCR using DNA from *Anabaena* as template and the primer pairs alr2817-34/alr2817-35 and alr2817-36/alr2817-37, respectively, with the alr2817-35 primer complementing the alr2817-36 primer (all oligodeoxynucleotide primers are listed in Table 1). The upstream and downstream DNA fragments were cloned in plasmid pMBL-T (Dominion MBL, Spain; the plasmids used or constructed in this work are described in Table 2) and sequenced, and the C.S3 gene cassette was inserted in the EcoRV site generated at the junction of the two cloned DNA fragments. The resulting construct was inserted into plasmid pRL278 [21] (see Table 2). The resulting plasmid, pCSL20, bears *Anabaena* DNA from the *hetC* locus with 3051 bp of DNA within *hetC* substituted by gene cassette C.S3.

**Table 1:** Oligodeoxynucleotide primers used in this work.

Name	Sequence 5′-3′
alr2817-13	GTTTCAAGAACGCAACG
alr2817-14	GATATCAGCTAAGTGGTGATAAAG
alr2817-34	TCTCTCTTGGGTGGGATTCT
alr2817-35	ATCGCCATTAGTTCCGATATCTTTGCAGCCCTCCCT
alr2817-36	AGGGAGGGCTGCAAAGATATCGGAACTAATGGCGAT
alr2817-37	CTCGCCTACTGTGCATTTGAGACT
alr2817-38	TGGTATCGGCTGAACAAA
alr2817-41	TGTCGCCACCCCAGTCATAAT
alr2817-42	CTTGAGGGCTTGAAAACTTTCTGGGTAGGAGTAGGACGA
alr2817-43	TCGTCCTACTCCTACCCAGAAAGTTTTCAAGCCCTCAAG
alr2818-13	CATGCTGAGCTCTGCTGGGTACAATCCTATGC
alr2818-8	AAAGTAAAGCTTACAAGCCAAGCATGAAAGTGGT
alr2818-9	CCGAAGGGTCTCACGCCATTATGAATAAAATC
alr2818-16	TAAATACTCTTGGGCTTGTGTTCT
alr2818-17	CTAGCTTCGGAGTTTTCTTTGAGT
alr2818-19	AAAGTAGGTACCACAAGCCAAGCATGAAAGTGGT
alr2820-1	CTTCTTGAGCTCTTACAGGAGAAGCCAGTCCAGGTT
alr5358-1	TTATACACCTTGCGTCCCTTCCTC
alr5358-2	CTCAACAGCTACATAGCGTGAAGCGCCGGT
alr5358-3	ACCGGCGCTTCACGCTATGTAGCTGTTGAG
alr5358-4	TGAAGTTCATCTCTGGCGCATTCC
asr2819-1	CTAAATCCCCTTTACGATATCATTCGAGAAGTTGCA
asr2819-2	TGCAACTTCTCGAATGATATCGTAAAGGGGATTTAG
asr2819-11	TTATTCCCGTCACTTACCACCATC
asr2819-12	TTCTAACTCAAGCACCACAAACTC
gfp-11	TCTGGTACCTTATTTGTATAGTTC
gfp-13	TTCTCCTTTGCTAGCACCTCCA
gfp-14	TGGAGGTGCTAGCAAAGGAGAA
rnpB-4	ACTCTTGGTAAGGGTGCAAAGGTG
rnpB-5	AACCATAGTTCCTTCGGCCTTGCT

**Table 2:** Plasmids used in this work.

Name	Description	Strain generated	Reference
pCSAL39	*sf-gfp* with N-terminal 4Gly linker-encoding sequence and BsaI site in a modified pMBL-T (Ap resistance substituted by C.K1 cassette)	-	A. López-Lozano and A. Herrero (unpublished)
pCSAM135	Contains a translational fusion between the 5′-terminal region of *sepJ* and the *gfp* gene		Flores *et al.* 2007
pCSL18	PCR product obtained with primers alr2817-34, alr2817-35, alr2817-36 and alr2817-37 (overlapping PCR), cloned in pMBL-T	-	This work
pCSL19	C.S3 inserted in EcoRV between the two DNA fragments of pCSL18	-	This work
pCSL20	pCSL19 digested with BamHI and XhoI, and cloned in pRL278	CSL3	This work
pCSL22	PCR product obtained with primers alr5358-1, alr5358-2, alr5358-3 and alr5358-4 (overlapping PCR), cloned in pMBL-T	-	This work
pCSL23	pCSL22 digested with BamHI and XhoI and cloned in pRL278	CSL7	This work
pCSL24	PCR product obtained with primers alr2817-38, alr2817-42, alr2817-43 and alr2817-41 (overlapping PCR), cloned in pMBL-T	-	This work
pCSL25	pCSL24 digested with SpeI and XbaI and cloned in pCSRO (XbaI)	CSL16	This work
pCSL68	pCSV3-derived plasmid containing the *mut2*-*gfp* gene	-	This work
pCSL69	PCR product obtained with primers alr2818-8 and alr2818-9, cloned in pCSAL39 digested with BsaI and HindIII	-	This work
pCSL70	pCSL69 digested with KpnI and cloned in pCSV3 digested with the same enzyme	CSL67	This work
pCSL111	PCR product obtained with primers asr2818-13, asr2819-1, asr2819-2 and alr2820-1 (overlapping PCR), cloned in pCSRO digested with SacI	CSL97	This work
pCSL123	PCR product obtained with primers alr2818-19, GFP-13, GFP-14 and GFP-11 (overlapping PCR), cloned in pCSV3 digested with KpnI	CSL107	This work
pCSM6	*hetC* PCR product obtained with primers alr2817-13 and alr2817-14 fused to *mut2-gfp* through an EcoRV site, cloned in PstI-digested pCSV3	CSM1	This work
pCSRO	Plasmid derived from pRL278, with Km^R^ gene substituted by C.S3	-	Merino-Puerto *et al.* (2010)
pCSV3	Plasmid derived from pRL500, with Ap^R^ gene substituted by C.S3	-	Olmedo-Verd *et al*. (2006)
pMBL-T	Commercial vector for cloning purposes	-	Dominion MBL
pRL278	Vector used for the positive selection of double recombinants in *Anabaena*	-	Black *et al*. (1993)

Template *Anabaena* DNA and overlapping primer pairs alr2817-38/alr2817-42 and alr2817-43/alr2817-41 were used to delete, specifically, the predicted peptidase domain of *hetC*, and the PCR product was cloned in plasmid pMBL-T, sequenced and transferred to pCSRO [22]. The plasmid produced, pCSL25, bears DNA from the *hetC* locus with a deletion of 378 bp from inside *hetC*.

To delete the *hetN* gene, template *Anabaena* DNA and overlapping PCR primer pairs alr5358-1/alr5358-2 and alr5358-3/alr5358-4 were used for amplification. The resulting DNA fragments were cloned in pMBL-T and sequenced, and then transferred to pRL278. The resulting plasmid was named pCSL23.

For deletion of ORF *asr2819*, template *Anabaena* DNA and overlapping primer pairs alr2818-13/asr2819-1 and asr2819-2/alr2820-1 were used for amplification. The PCR product was cloned in pCSRO, and sequenced, producing plasmid pCSL111.

Plasmids pCSL20, pCSL23, pCSL25, and pCSL111 were transferred to *Anabaena* by conjugation, performed as described [23]. Exconjugants were selected by their resistance to Sm and Sp, or to Nm, and double recombinants were then selected by their resistance to sucrose (sucrose sensitivity is conferred by the *sacB* gene present in the vector portion of pRL278 or pCSRO), and the chromosome structure in the altered region was tested by PCR.

For construction of a fusion of *hetC* to the *gfp-mut2* gene, a DNA fragment was amplified using *Anabaena* DNA as template and primer pair alr2817-13/alr2817-14. The PCR product was cloned in pMBL-T, and afterwards in pCSAM135, which bears the *gfp-mut2* sequence [24], and the resulting *hetC*-*gfp-mut2* construct was transferred to the mobilizable vector pCSV3 [25] to produce plasmid pCSM6. For construction of a *hetP-sf-gfp* fusion, the alr2818-8/alr2818-9 primer pair was used, and the resulting PCR product was cloned in plasmid pCSAL39 (which includes a sequence encoding a four-Gly linker preceded by a BsaI site just before the *sf-gfp* sequence). The resulting construct was cloned in pCSV3 producing plasmid pCSL70. For construction of a *hetP-gfp*-*mut2* fusion, two DNA fragments were amplified by PCR. One contained the *hetP* gene and was amplified from plasmid pCSL70 with primer pair alr2818-19/gfp-13. The other contained the *gfp*-*mut2* gene and was amplified from plasmid pCSL68 with the primer pair gfp-14/gfp-11. The fragments produced were joined by overlapping PCR, and the product was cloned in pCSV3 rendering plasmid pCSL123.

Plasmids pCSM6, pCSL70, and pCSL123 were transferred to *Anabaena* sp. by conjugation. Clones resistant to Sm and Sp, which had integrated the plasmid in the resident genomic locus by a single recombination event, were selected and their genomic structure was tested by PCR.

### Northern blot analysis, qRT-PCR, Hgl determination and nitrogenase activity

Isolation of total RNA from different strains of *Anabaena* and northern blot analysis was performed as described [26]. Retrotranscription was performed with a QuantiTect Reverse Transcription kit (Qiagen) and the degenerated oligonucleotides “Random hexamer primers” (Bioline). Real-time PCR was performed in an iCycler iQ (BioRad) with the SensiFAST SYBR & Fluorescein kit using 1/10 volumes of the cDNA resulting from the retrotranscription step and primer pairs alr2818-16/alr2818-17, asr2819-11/asr2819-12, and rnpB-4/rnpB-5. Gene-expression ratios and statistical parameters were calculated with the REST 2009 software (http://rest-2009.gene-quantification.info).

Lipid analysis was performed as in [26]. Nitrogenase activity was determined under oxic and anoxic conditions in filaments incubated for 24 or 48 h in bubbled cultures without combined nitrogen and without antibiotics [26].

### Microscopy

For light microscopy, filaments grown in BG11_0_ + ammonium medium (in the presence of antibiotics when appropriate) were harvested, washed with nitrogen-free (BG11_0_) medium and incubated for 24 h in bubbled cultures at 30°C in the light. At least 300 cells or 100 intervals were counted for each strain in each of two to six independent experiments. Dividing cells were counted as two cells. Cell suspensions were mixed (1∶10) with a 1% Alcian Blue (Sigma) solution [5]. Because Alcian Blue stains the polysaccharide (Hep) layer of the heterocyst envelope, the stained cells, to which we will refer collectively as (pro)heterocysts, comprise mature heterocysts and proheterocysts. In *Anabaena*, a proheterocyst is an intermediate between a vegetative cell and a heterocyst, which differs in shape and granularity from vegetative cells, and a mature heterocyst is a cell capable of aerobic fixation of N_2_ (see [27]).

Microscopy was performed with a confocal microscope as in [5], treating the images with the LAS AF Software (Leica), or with a Leica DM6000B fluorescence microscope and an ORCA-ER camera (Hamamatsu) using an FITC L5 filter (excitation, band-pass [BP] 480/40 filter; emission, BP 527/30 filter). BlindDeblur deconvolution of 3D images was made with the LAS AF Leica software.

## Results

### Deletion of *hetC* in *patS* and *hetN* backgrounds

**Figure 1:**
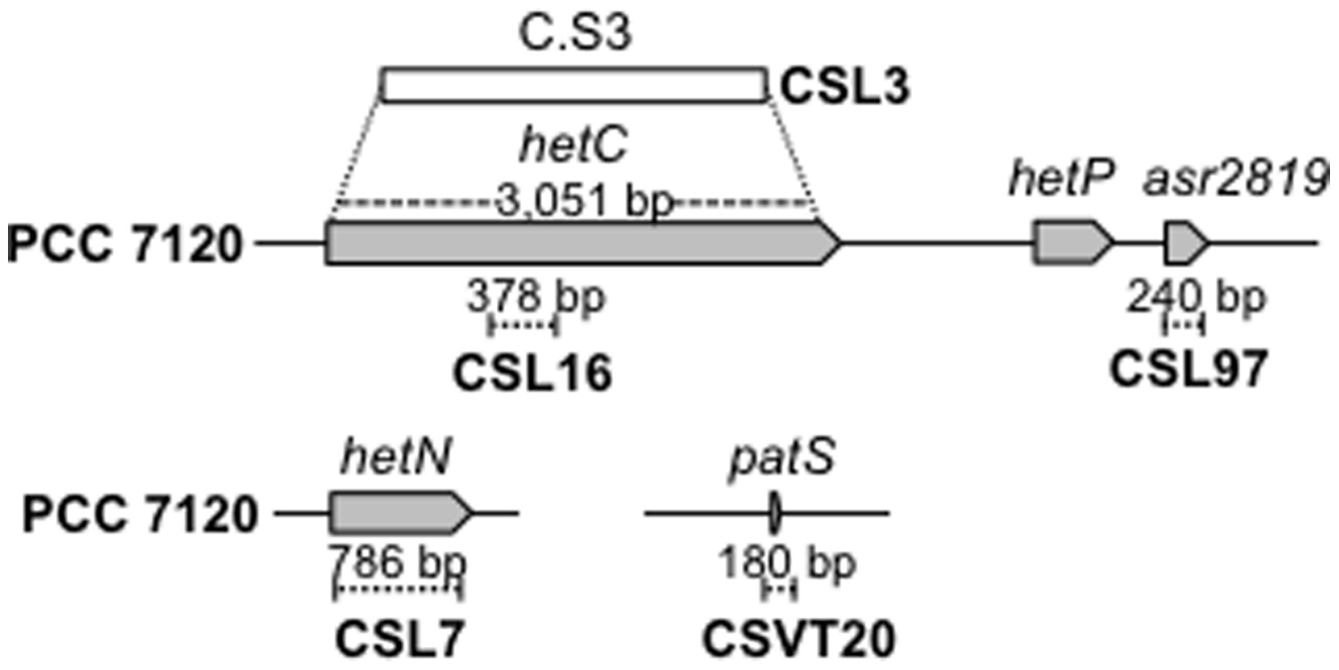
Schematics of the *hetC*, *hetN* and *patS* genomic regions in *Anabaena* sp. strain PCC 7120 and mutant derivatives. The gene map is from [38]. The *Anabaena* genes are represented with grey arrows, the deleted portions (of the specified sizes) with dashed segments, and C.S3 gene-cassette insertion with a white bar. The names of the resulting mutant strains in the wild-type genetic background are indicated.

**Figure 2:**
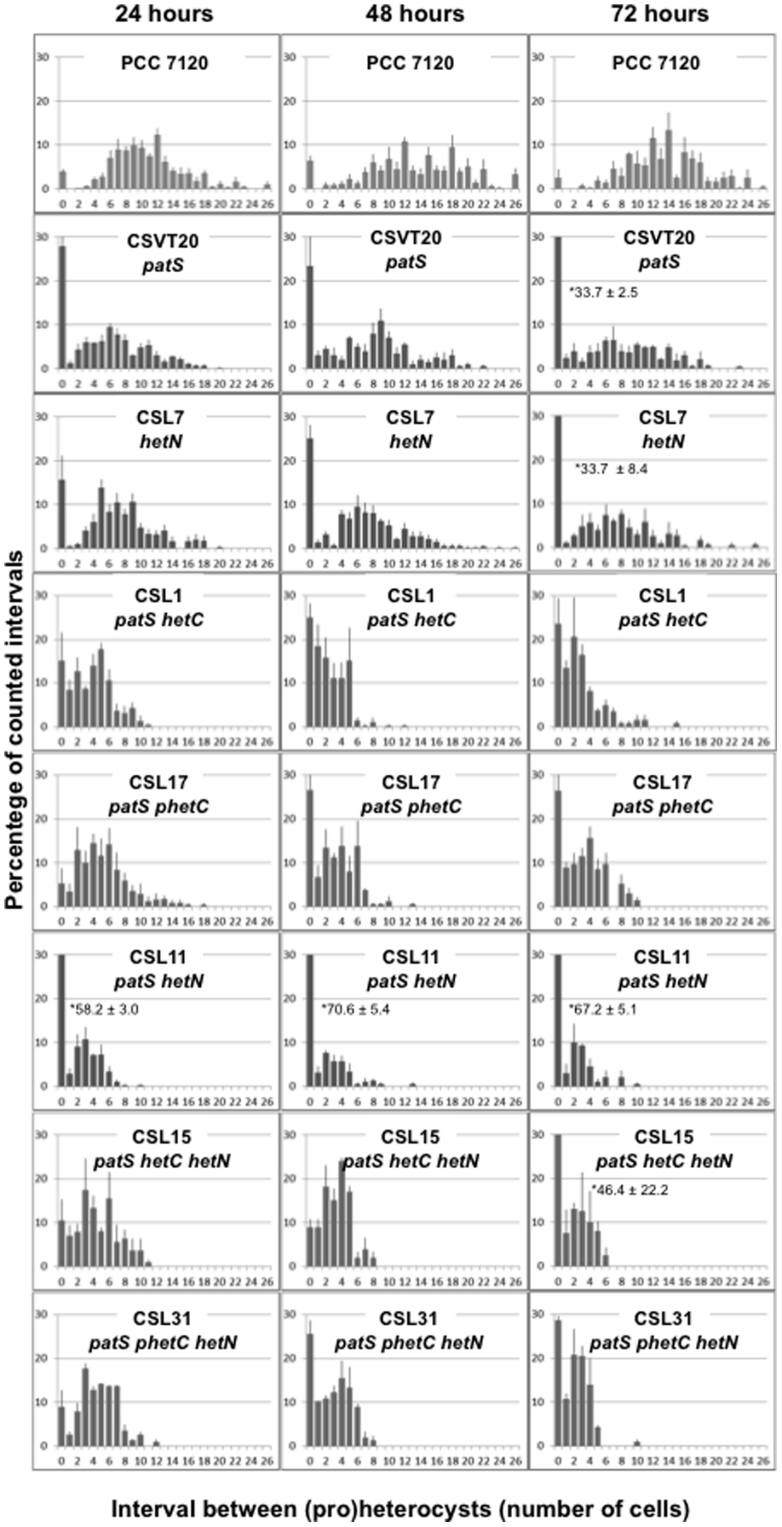
Heterocyst distribution in *Anabaena* mutant strains. Filaments from bubbled, ammonium-supplemented cultures of the indicated strains were washed three times with BG11_0_ medium, resuspended in BG11_0_ and incubated under the same culture conditions for 24, 48 or 72 h (as indicated). Cells were counted after staining with Alcian Blue. Data are the mean and standard deviation of the mean of two to six independent experiments (see Materials and Methods).

**Figure 3:**
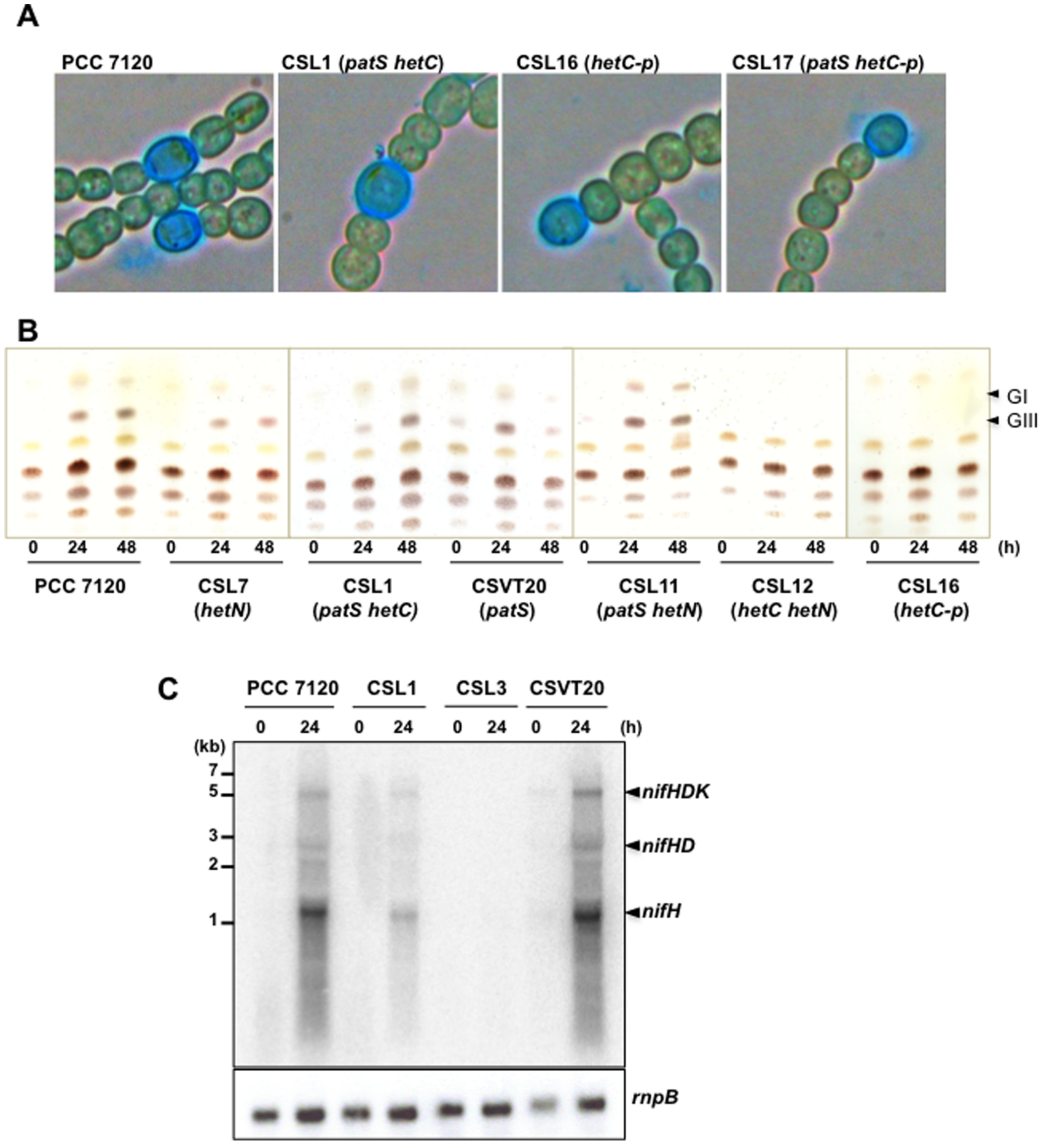
Microscopy, lipids and *nifHDK* expression in *Anabaena* mutant strains. Filaments from bubbled, ammonium-supplemented cultures of the indicated strains were washed three times with BG11_0_ medium, resuspended in BG11_0_ medium and incubated under the same culture conditions for the times indicated in h. (A) Samples taken at 24 h were stained with Alcian Blue prior to being photographed under a light microscope. (B) Lipids (GI and GIII are heterocyst envelope glycolipids) were isolated and separated by TLC. (C) Total RNA was isolated and used in northern blot analysis with a probe of the *nifH* gene or, as a loading and transfer control, the *rnpB* gene. A size standard is indicated at the left and the observed transcripts at the right.

To test a possible functional relationship between the HetC protein required for heterocyst differentiation and the differentiation negative regulatory factors PatS and HetN, we generated mutant strains of *Anabaena* lacking one, two or the three of these factors (see schemes of the genomic regions in Fig. 1). Strain CSVT20 (Δ*patS*) has been described previously [5]. The mutants were analyzed in terms of frequency and distribution of cells stained with Alcian Blue and the presence or absence of heterocyst-specific glycolipids (Hgl). The data are presented in Table 3 and Figures 2 and 3.

**Table 3:** Spatial pattern of heterocysts in *Anabaena* sp. PCC 7120 mutant strains.

Strain	Genotype	Time (h)	Percentage heterocysts	Contiguous heterocysts	Mean interval
PCC 7120		24	10.4±0.4	3.9±0.6	10.5±0.7
		48	8.6±0.4	6.4±1.0	13.1±0.8
		72	7.5±0.7	2.5±1.9	13.3±0.8
CSL3	*hetC*	24	0.03±0.02	-	-
		48	0.00±0.0	-	-
		72	0.00±0.0	-	-
CSL16	*hetC-p*	24	1.1±0.7	-	-
		48	2.8±1.6	-	-
		72	1.4±1.0	-	-
CSVT20	*patS*	24	18.6±2.2	27.8±8.0	5.5±0.7
		48	17.2±1.0	23.4±6.7	6.8±0.4
		72	17.6±1.0	33.7±2.5	6.0±0.6
CSL7	*hetN*	24	14.3±1.0	15.7±5.5	6.8±0.1
		48	13.9±0.6	25.0±3.1	6.2±0.2
		72	17.7±1.0	33.7±8.4	5.6±1.2
CSL1	*patS hetC*	24	16.4±1.5	15.1±6.4	3.8±0.4
		48	29.4±1.8	25.0±3.2	2.3±0.2
		72	33.0±2.7	23.7±5.7	2.7±0.2
CSL17	*patS hetC-p*	24	18.5±3.4	5.4±3.4	5.2±0.7
		48	25.2±2.6	26.6±6.0	3.0±0.5
		72	24.7±3.1	26.5±4.0	3.1±0.2
CSL11	*patS hetN*	24	42.7±4.8	58.2±3.0	1.5±0.2
		48	62.5±10.0	70.6±5.4	1.1±0.3
		72	65.0±6.7	67.2±5.1	1.1±0.02
CSL12	*hetC hetN*	24	2.5±0.8	-	-
		48	2.1±0.8	-	-
		72	1.8±0.8	-	-
CSL30	*hetC-p hetN*	24	2.1±1.3	-	-
		48	1.2±0.6	-	-
		72	0.8±0.6	-	-
CSL15	*patS hetC hetN*	24	28.8±2.0	10.5±4.8	4.3±1.0
		48	36.5±0.4	8.9±1.9	3.3±0.1
		72	57.3±12.7	46.4±22.2	1.7±0.6
CSL31	*patS hetC-p hetN*	24	20.2±1.1	8.9±3.8	4.5±0.3
		48	34.8±7.8	25.5±3.2	2.8±0.1
		72	43.5±6.2	28.6±1.0	2.0±0.03
CSL101	*patS hetC-p* (*patS8*)	24	1.6±0.7	-	-
CSL102	*patS hetC-p* (*patS17*)	24	1.8±0.3	-	-
CSL67	*hetP-sfgfp*	24	11.5 ±0.8	9.1±2.5	8.6±0.6
		48	11.4±0.6	20.5±0.4	9.8±0.4
		72	11.0±0.6	14.7±1.2	11.1±0.9
CSL68	*hetP-sfgfp* *hetC-p*	24	12.1±1.8	13.4±2.4	7.7±0.4
		48	11.5±1.2	13.7±5.6	9.7±0.3
		72	14.7±1.8	19.4±3.9	10.4±0.6
CSL69	*hetP-sfgfp* *patS*	24	25.5 ±6.3	26.3±6.9	5.7±1.2
		48	27.0±9.6	19.8±0.8	7.5±0.2
		72	14.9±2.7	21.2±3.1	8.6±1.1
CSL70	*hetP-sfgfp* *hetN*	24	17.6±1.3	29.2±1.0	4.8±0.3
		4872	22.9±3.116.1±0.2	47.3±0.0144.4±4.8	4.4±0.15.2±0.1
CSL97	*asr2819*	24	13.8±2.6	1.8±0.9	8.2±0.6
		48	12.5±1.1	4.9±0.9	8.2±0.7
		72	15.8±0.4	5.5±1.3	10.5±1.1
CSL98	*asr2819* *hetC-p*	24	1.2±0.1	-	-
		48	3.0±1.1	-	-
		72	1.1±0.4	-	-

Filaments were grown with ammonium and incubated for the indicated times in the absence of combined nitrogen. Heterocyst frequency (as percentage of total cells), the mean size of vegetative cell intervals between heterocysts, and the percentage of contiguous heterocysts (interval size  = 0, percentage of total intervals) were calculated (for the strains included in Figs. 2, 6 and 7 values are from the data in the figures).

As is the case with other previously described mutants of *hetC*, strain CSL3 (a *hetC* single mutant) did not produce (pro)heterocysts. As previously described, the *patS* single mutant, strain CSVT20, shows a Mch phenotype with more heterocysts and shorter vegetative-cell intervals between heterocysts than in the wild-type strain [5]. However, in contrast to previous reports describing that the Mch phenotype of a *patS* mutant was alleviated at 72 h after N step-down [4], strain CSVT20 maintained the increased frequency of single and contiguous heterocysts at this time (Table 3, Fig. 2). In the *patS hetC* double mutant, strain CSL1 (see Fig. 3A), (pro)heterocyst frequency was similar to that of the *patS* single mutant at 24 h, and somewhat higher at 48 (1.67-fold) and 72 (1.87-fold) h after N step-down. In strain CSL1, the mean interval size of vegetative cells between (pro)heterocysts was smaller than in the *patS* single mutant, and the percentage of contiguous (pro)heterocysts (interval size  = 0) was lower (at 24 and 72 h) than in CSVT20. Indeed, aside from the contiguous (pro)heterocysts, the distribution of interval sizes is notably different in strains CSL1 and CSVT20, with more tendency to short intervals different from zero in the former (Fig. 2). Finally, Hgls, which are not detectable in the *hetC* mutant (not shown), were present in strain CSL1 as well as in CSVT20 (Fig. 3B). Therefore, the requirement for a functional HetC product can be overridden by mutation of *patS* at least up to the stage of the formation of proheterocysts with Hep and Hgl layers.

Strain CSL7 (*hetN*) showed more heterocysts, shorter intervals and more contiguous heterocysts than the wild type at all time-points, with the percentage of contiguous heterocysts increasing during incubation in the absence of combined nitrogen. These observations differ from those in a previous report of the effects of down-regulation of *hetN*, expressed from the heterologous P*_petE_* gene promoter, which led to Mch at 48 h but not earlier [28]. The differences between our results and those reported for other *patS* and *hetN* mutants could be due to differences in the genetic structure of the compared strains. Strain CSL12 (*hetN hetC*) was also able to form (pro)heterocysts, although at frequencies considerably lower than in the *hetN* single mutant or the wild type (Table 3), and, like the *hetC* single mutant, lacked Hgl (Fig. 3B). Thus, deletion of *hetN* does not compensate for the lack of HetC, although it provides a capacity to proceed slightly with differentiation that is not observed in the *hetC* single mutant.

Finally, we generated strain CSL11, in which *patS* and *hetN* were deleted (in the *patS hetN* double mutant described previously [12], *hetN* was conditionally down-regulated), and strain CSL15, a triple *hetC patS hetN* deletion mutant. In strain CSL11, the frequencies of (pro)heterocysts and contiguous heterocysts were considerably higher, and the mean interval shorter, than in the *patS* or *hetN* single mutants at all time points. As might be expected, like the *patS* and *hetN* single mutants, strain CSL11 produced Hgl (Fig. 3B). In strain CSL15, the percentage of (pro)heterocysts was considerably higher than in the *hetC patS* double mutant (strain CSL1), reaching ca. 57% of the total cells after 72 h in the absence of combined nitrogen. In CSL15 the mean vegetative cell interval between heterocysts was shorter than in CSL1 (0.6 times) at 72 h, and the percentage of contiguous heterocysts was lower at 24 and 48 h but about twice at 72 h. Thus, inactivation of *hetN* led to a further increase in the differentiation capacity relative to the *hetC patS* mutant. In comparison to CSL11 (*patS hetN*), CSL15 (*hetC patS hetN*) had fewer heterocysts (0.67-fold at 24 h, 0.58-fold at 48 h and 0.88-fold at 72 h), many fewer contiguous heterocysts (0.18-fold, 0.13-fold and 0.69-fold, respectively) and more short intervals different from zero, especially at 24 and 48 h (Table 3, Fig. 2). Thus, in the absence of both PatS and HetN, the lack of HetC appears again to shift the Mch pattern to more individual heterocysts.

### An altered *hetC* gene version

**Figure 4:**
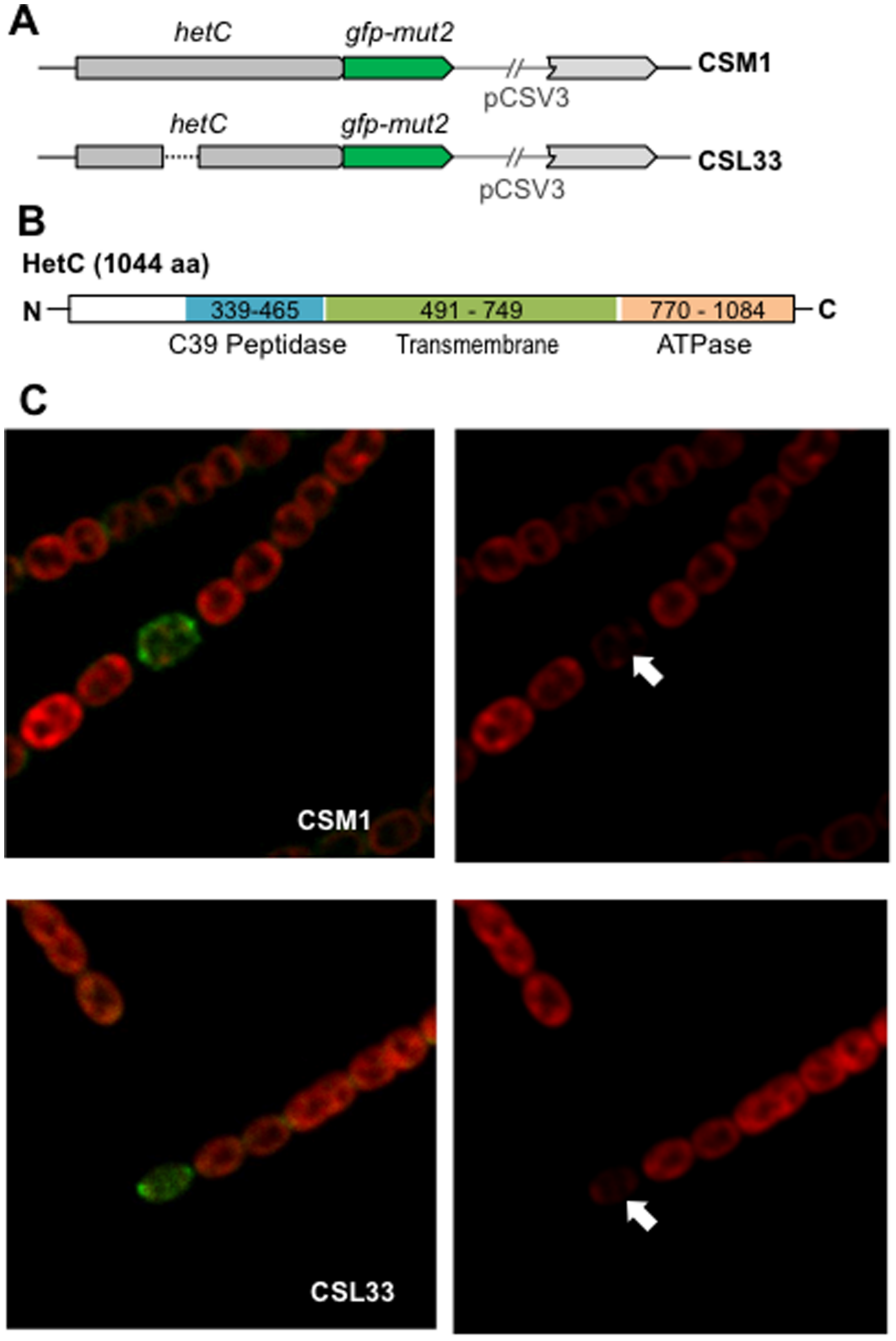
Localization of HetC-GFP. (A) Scheme of the genomic *hetC* region of strains CSM1 (expressing a HetC-GFP-mut2 fusion protein) and CSL33 (expressing a HetC-p-GFP-mut2 fusion protein). The pCSV3 vector portion integrated in the *hetC* locus is represented as a thin line. (B) Scheme of the putative domains of the HetC protein. (C) Confocal microscopy of filaments of strains CSM1 and CSL33 grown in bubbled, ammonium-supplemented medium and incubated for 24 h in medium containing no combined nitrogen. Cyanobacterial autofluorescence (red) is shown in the right-hand images, and merged autofluorescence and GFP fluorescence (green) in the left-hand images. Heterocysts (indicated with white arrows) are identified by their greatly diminished autofluorescence.

The *hetC* gene product includes a putative peptidase domain of the C39 family (InterPro, IPR005074; residues 339-465) (see Fig. 4B), which according to topological predictions would be located in the cytoplasm (Toppred). To test the functionality of this domain of HetC, mutant strains were generated that bore a *hetC* gene (*hetC-p*) that encodes a HetC version that lacks residues 338–463 that comprises the putative peptidase domain (see Fig. 1). The strain bearing this construct in the wild-type background (CSL16) was severely impaired in heterocyst differentiation although, in contrast to the mutant lacking the whole *hetC* gene (CSL3), produced a low percentage of (pro)heterocysts (Table 3) that could be stained with Alcian blue (Fig. 3A) but bore no Hgl (Fig. 3B). On the other hand, the percentage and distribution of (pro)heterocysts in strains bearing the *hetC-p* version in a *patS* background (CSL17), in a *hetN* background (CSL30) or in a *patS hetN* background (CSL31) followed a trend similar to the strains bearing the full *hetC* deletion in the same backgrounds (strains CSL1, CSL12 and CSL15, respectively) (Table 3, Fig. 2, see also Fig. 3A). Thus, the region that contains the putative peptidase domain appears necessary for proper HetC function. However, the percentage of contiguous (pro)heterocysts at 24 h was ca. 2.8 times higher in CSL1 than in CSL17, and at 48 and 72 h ca. 2.9 and 1.6 times higher, respectively, in CSL15 than in CSL31.

To test whether the function of the peptidase domain of HetC is related to processing of PatS, we expressed from the *patS* promoter in the *patS* locus, in the *hetC-p patS* background, *patS* minigenes that encode the full 17-residue peptide (strain CSL102) or a peptide consisting of a Met residue followed by the PatS eight C-terminal residues (CSL101) [5] as the only *patS* version. In both strains, the (pro)heterocyst frequency was comparable, ca. 1.8% and 1.6% for CSL102 and CSL101, respectively, at 24 h (Table 3), and similar to that of strain CSL16 (*hetC-p*), whereas the parental strain CSL17 (*patS hetC-p*) had 18.5% of heterocysts at 24 h. Thus, both *patS* minigenes complemented the lack of *patS* in a *hetC-p* background.

### Nitrogenase activity and diazotrophic growth in *hetC*, *patS* and *hetN* mutants

Nitrogenase activity, determined under both oxic and anoxic assay conditions, and diazotrophic growth were investigated in some of the mutants described above (Table 4; see Materials and Methods for details). The two *hetC* single-mutant strains, CSL3 (*hetC*) and CSL16 (*hetC-p*), exhibited negligible levels of nitrogenase activity under oxic or anoxic conditions. Strains CSL1 (*hetC patS*) and CSL17 (*hetC-p patS*) developed appreciable levels of nitrogenase activity under anoxic conditions, although those levels were much lower than in the wild type. Notably, in contrast to CSL3, significant expression of *nifHDK* took place in CSL1, although at 24 h after N step-down the transcript levels were lower than those observed in the wild type or the *patS* single mutant (Fig. 3C). However, as is the case for CSL3, strain CSL1 did not grow diazotrophically (Table 4). Thus, the lack of *patS* does not allow heterocyst function in the absence of a functional *hetC* gene. Strains CSL12 (*hetC hetN*) and CSL30 (*hetC-p hetN*) exhibited negligible nitrogenase activity under any tested condition and they were incapable of diazotrophic growth (Table 4). Thus, deletion of *hetN* appears unable to compensate for inactivation of *hetC*. In the *hetC patS hetN* (strain CSL15) or *hetC-p patS hetN* (CSL31) triple mutants, appreciable nitrogenase activity developed under anoxic conditions, as was the case in the *patS hetN* double mutant (CSL11). None of these strains was capable of effective diazotrophic growth (Table 4).

**Table 4:** Nitrogenase activity and diazotrophic growth in *Anabaena* sp. PCC 7120 mutant strains.

Strain	Genotype	Time (h)	Nitrogenase activityOxic Anoxic		Diazotrophic growth
PCC 7120		24	16.0±3.6 (4)	17.7±1.1 (12)	YES
		48	8.3±2.4 (6)	11.6±0.7 (7)	
CSL3	*hetC*	24	0.0±0.0 (3)	0.1±0.03 (7)	NO
		48	0.1±0.1 (4)	0.1±0.1 (3)	
CSL16	*hetC-p*	24	0.0±0.0 (2)	0.0 (1)	NO
		48	0.1±0.1 (3)	0.0 (1)	
CSVT20	*patS*	24	3.2 (1)	12.7±1.0 (6)	YES
		48	1.1±0.2 (2)	7.8±0.7 (2)	
CSL7	*hetN*	24	3.6 (1)	10.5±3.1 (3)	YES
		48	2.1±0.7 (2)	6.1±0.9 (3)	
CSL1	*patS hetC*	24	0.0±0.02 (2)	0.8±0.5 (6)	NO
		48	0.0±0.01 (2)	0.4±0.3 (4)	
CSL17	*patS hetC-p*	24	0.1±0.04 (3)	0.2±0.1 (3)	NO
		48	0.7±0.4 (4)	1.3±0.8 (4)	
CSL11	*patS hetN*	24	0.0 (1)	1.7±1.4 (3)	NO
		48	0.03±0.04 (2)	0.5±0.4 (4)	
CSL12	*hetC hetN*	24	0.0 (1)	0.0 (1)	NO
		48	0.01±0.1 (2)	0.1±0.03 (2)	
CSL30	*hetC-p hetN*	24	0.0 (1)	0.0±0.0 (2)	NO
		48	0.02±0.02 (2)	0.1±0.1 (2)	
CSL15	*patS hetC* *hetN*	24	0.1±0.1 (2)	0.2±0.1 (3)	NO
		48	0.1±0.1 (2)	2.2±1.8 (3)	
CSL31	*patS hetC-p hetN*	24	0.1±0.03 (2)	0.3±0.1 (3)	NO
		48	0.8±0.4 (3)	2.4±0.7 (4)	
CSL67	*hetP-sfgfp*	24	10.2±3.3 (2)	22.1 (1)	YES
		48	8.2±3.0 (2)	14.9 (1)	
CSL68	*hetP-sfgfp* *hetC-p*	24	12.5 (1)	14.0 (1)	YES
		48	4.3±0.02 (2)	7.9±1.8 (2)	
CSL69	*hetP-sfgfp* *patS*	24	1.4 (1)	7.6 (1)	YES
		48	1.6 (1)	7.4 (1)	
CSL70	*hetP-sfgfp* *hetN*	24	6.6 (1)	9.8 (1)	YES
		48	3.8 (1)	9.2 (1)	

Nitrogenase activity was determined in ammonium-grown filaments incubated in the absence of combined nitrogen for 24 or 48 h and expressed in µmol ethylene produced (mg Chl)^−1^ h^−1^. Data are the mean and standard deviation of the mean (number of independent experiments indicated in parenthesis). Diazotrophic growth was tested in solid BG11_0_ medium.

### HetC localization

It has been described that a *hetC-gfp* transcriptional fusion is expressed most strongly in proheterocysts and heterocysts [16]. Predictions of the topology of the putative HetC protein (SOSUI) identified a membrane domain with six transmembrane helices (residues 491–749) between the putative peptidase and ATPase stretches (Fig. 4B). To study the subcellular localization of this protein, *Anabaena* derivatives producing a HetC-GFP (strain CSM1) or a HetC-p-GFP (Δpeptidase HetC-GFP) (strain CSL33) fusion protein as the only HetC version were generated (Fig. 4A). In these constructs, the GFP is added to the C-terminus of the corresponding HetC protein. Upon N step-down, fluorescence above the background was detected after ca. 6 h, located throughout the (pro)heterocyst surface but especially concentrated within the polar region of the heterocyst, apparently in the narrow area corresponding to the heterocyst neck (Fig. 4C). Because strain CSM1 expresses HetC fused to the *gfp*-*mut2*-encoded GFP, which only folds efficiently in the cytoplasm, our results support that the C terminus of HetC is located in the cytoplasm. No difference in GFP fluorescence was apparent between strains CSM1 and CSL33 (Fig. 4C), indicating that the putative peptidase stretch of HetC does not appreciably influence the production and sub-cellular localization of HetC.

### HetP localization and effects of over-expression of *hetP*

**Figure 5:**
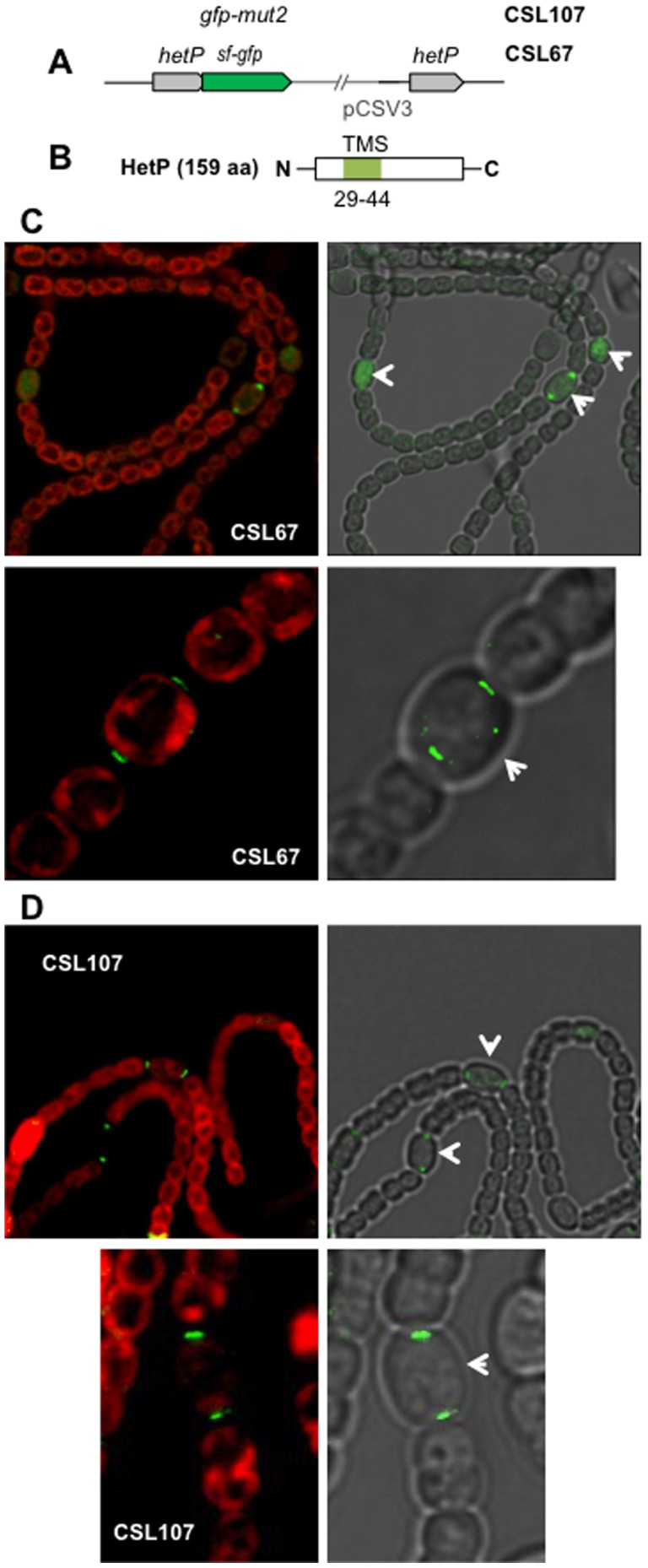
Localization of HetP-GFP. (A) Scheme of the *hetP* genomic region of strains CSL67 (HetP-sf-GFP fusion protein) and CSL107 (HetP-GFP-mut2 fusion protein). The pCSV3 vector portion integrated in the *hetP* locus is represented as a thin line. (B) Scheme of the HetP protein showing a predicted transmembrane segment (TMS). (C) Confocal microscopy of filaments of strain CSL67 grown in BG11_0_ solid medium (upper part) or deconvoluted fluorescence microscopy image of filaments of strain CSL67 grown in bubbled ammonium-supplemented medium and incubated for 40 h in medium containing no combined nitrogen (lower part). (D) Fluorescence microscopy of filaments of strain CSL107 grown in BG11_0_ solid medium. Merged images of autofluorescence and GFP fluorescence are shown at the left side, and of bright field and GFP fluorescence at the right side. Heterocysts and some proheterocysts are indicated with white arrows.

**Figure 6:**
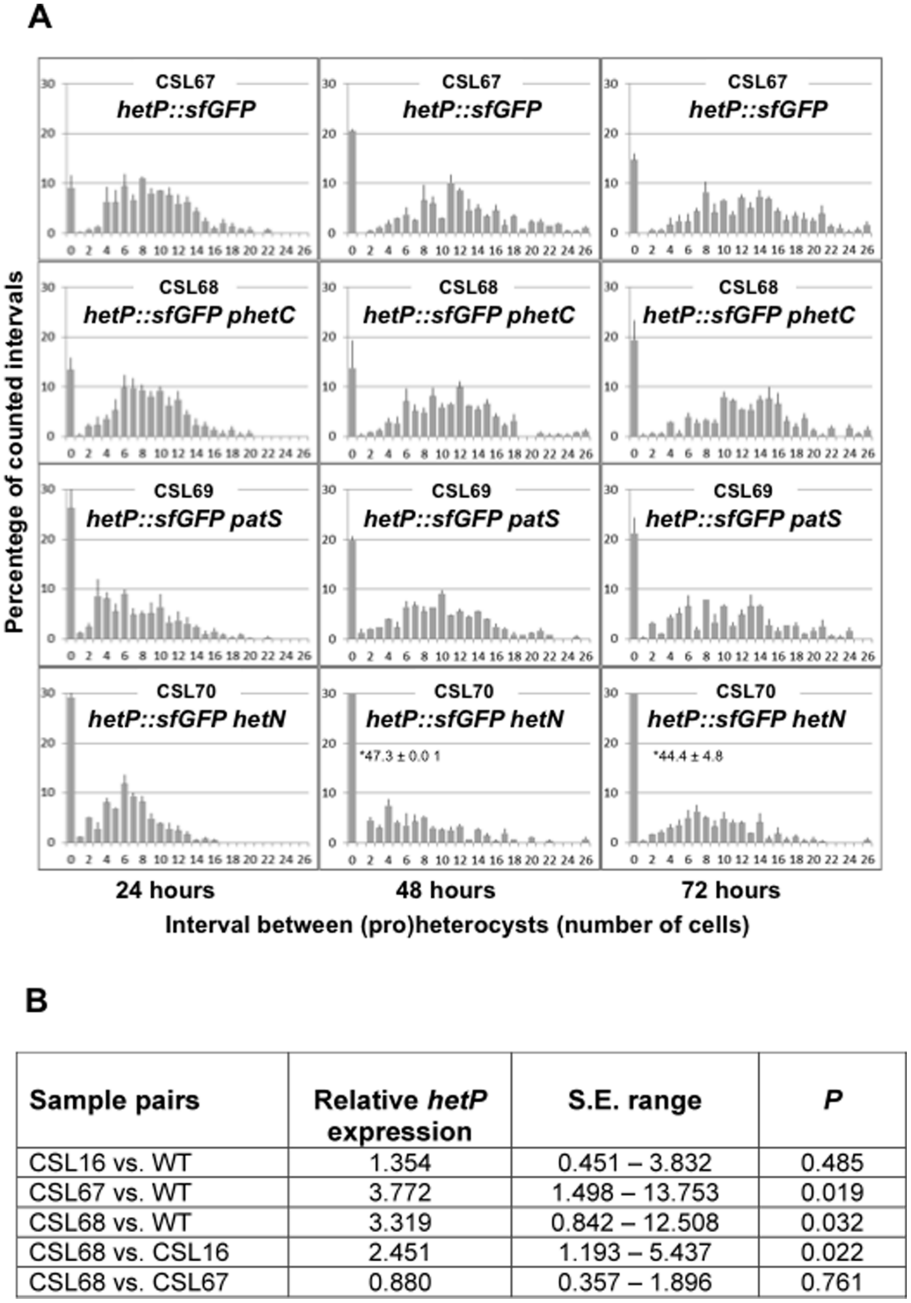
Heterocyst distribution and *hetP* expression levels in *Anabaena* mutant strains altered in *hetP*. (A) Heterocyst distribution in the indicated strains grown in bubbled cultures with ammonium and incubated for the indicated times in the absence of combined nitrogen under culture conditions (see legend to Fig. 2 for details). (B) Ratios of the expression levels of *hetP* of the indicated strains 18 h after N step-down, measured by qRT-PCR normalized to the *rnpB* gene. S.E. range indicates the “standard error change” and *P* (the hypothesis test P) represents the probability that the difference between the sample and control groups is due only to chance [39]. Data are the mean of two independent experiments.

Previously, a transcriptional fusion of *hetP* to *luxAB* was reported to increase 2.5-fold in expression 6 h after N step-down [17], and a P*_hetP_-gfp* fusion was shown to produce higher fluorescence in heterocysts than in vegetative cells at 24 h [18]. We have constructed strains bearing a *hetP-gfp-mut2* or *hetP-sf-gfp* gene fusion that encode, respectively, a HetP protein C-terminally fused to conventional GFP (strain CSL107) or to sf-GFP [29] (strain CSL67). These strains bear the fusion gene preceding the inserted pCSV3 plasmid vector and a native *hetP* copy in the chromosome (Fig. 5A; see Materials and Methods). The *hetP-sf-gfp* construct was also introduced into the *hetC-p*, the *patS* and the *hetN* mutant backgrounds producing strains CSL68, CSL69 and CSL70, respectively. (Pro)heterocyst frequency and distribution was studied in each of those strains (Table 3, Fig. 6A; for comparison, see also Fig. 2). In general terms, strains CSL67, CSL69 and CSL70 exhibited (pro)heterocyst patterns similar to those of their respective parental strains (strains PCC 7120, CSVT20 and CSL7), although with a tendency to increase heterocyst frequency and the frequency of doublets (considerably higher in strain CSL67 than in PCC 7120), and to decrease the mean interval size between heterocysts. However, in contrast to strain CSL16 (*hetC-p*), which presented a very low (pro)heterocyst frequency (Table 3), strain CSL68 showed a frequency of heterocysts and mean interval size similar to those in the wild type. The frequency of contiguous heterocysts in strain CSL68 was much higher (7.8 times at 72 h) than in the wild type.

Nitrogenase activity under oxic and anoxic conditions was comparable in strains CSL67, CSL69 and CSL70 and their respective parental strains, and those three mutant strains were capable of diazotrophic growth (Table 4). In contrast to its parental strain, CSL16, which showed negligible activity levels, strain CSL68 showed high levels of oxic and anoxic nitrogenase activity, and indeed it was capable of diazotrophic growth (Table 4). Because the *hetP* gene influences heterocyst differentiation positively [17], [18], the above results suggest that the HetP-sf-GFP fusion protein is functional. The relative expression levels of *hetP* (or *hetP-sf-gfp*) were studied by qRT-PCR in strains PCC 7120, CSL16 (*hetC-p*), CSL67 (*hetP-sf-gfp* in wild-type background) and CSL68 (*hetP-sf-gfp* in *hetC-p* background). Results included in Figure 6B indicate that 18 h after N step-down the *hetP* transcript levels were ca. 3- to 4-fold higher in strains CSL67 and CSL68 than in their respective parental strains. Thus, the increased differentiation in strain CSL67 with respect to the wild type could result from the increased expression of a functional *hetP* gene. Moreover, the comparison of (pro)heterocyst pattern and *hetP* expression in strains CSL16 and CSL68 indicates that over-expression of *hetP* compensates for the lack of a functional *hetC* gene.

Fluorescence from sf-GFP was tracked in strains CSL67, CSL68, CSL69 and CSL70. In CSL67 (*hetP-sf-gfp* in a wild-type background), fluorescence was located throughout the cell area in proheterocysts and focalized near the cell poles in mature heterocysts (Fig. 5C). Deconvolution analysis of the images from mature heterocysts showed the GFP fluorescence in the cytoplasm (Fig. 5C, see merged GFP/bright-field image) but external to the chlorophyll fluorescence (Fig. 5C, see merged GFP/autofluorescence image). Localization of fluorescence was similar in strains CSL68, CSL69 and CSL70 (not shown). Similar *sf-gfp* expression levels were detectable by qRT-PCR in strains CSL67 and CSL68 (not shown). In strain CSL107 bearing a *hetP-gfp-mut2* gene version, GFP fluorescence was similar to that in CSL67 (Fig. 5D), suggesting that HetP-GFP is folded in the cytoplasm.

### Mutation of *asr2819*

**Figure 7:**
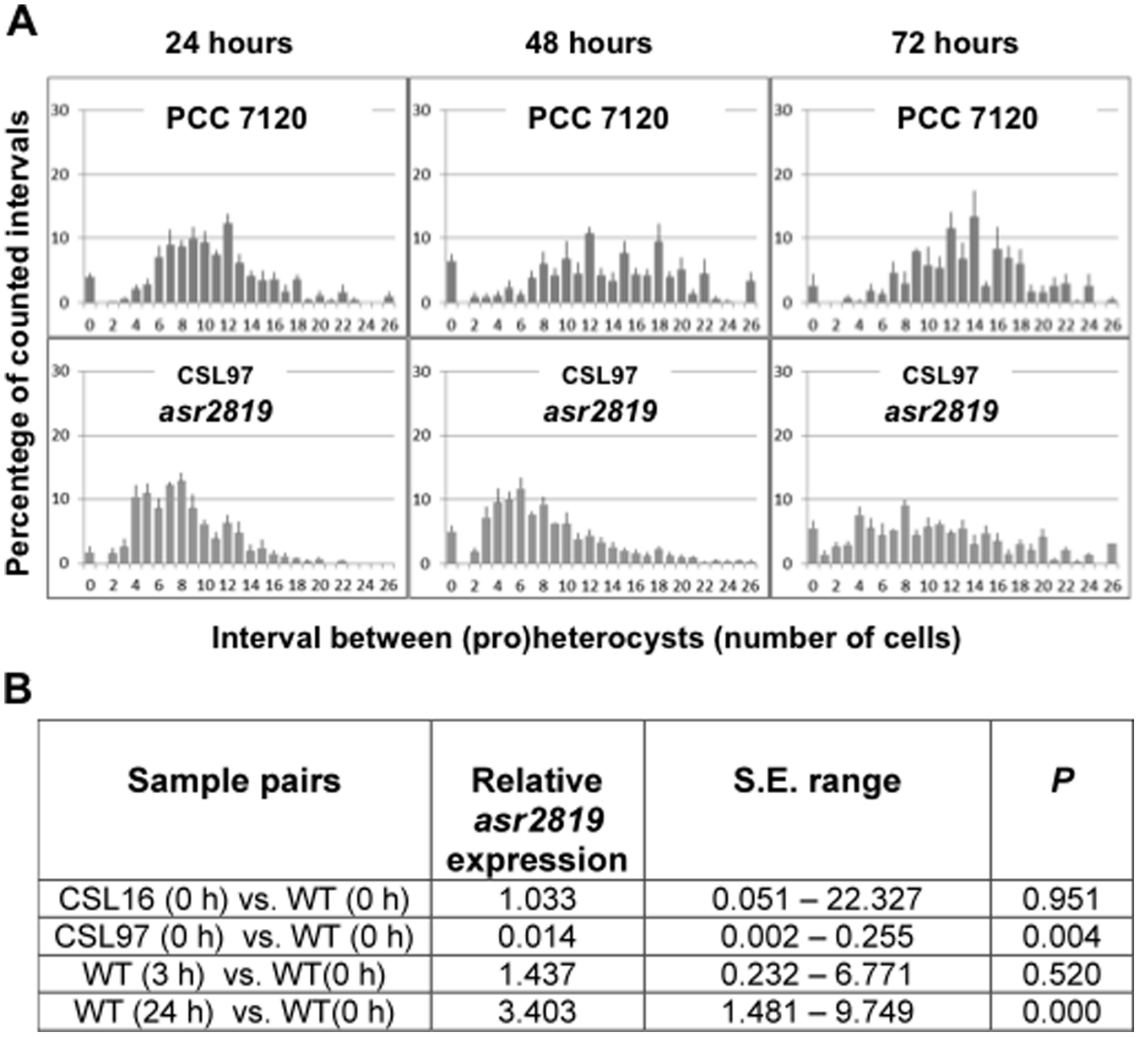
Heterocyst distribution and *asr2819* expression levels in *Anabaena* sp. strains PCC 7120 and CSL97. (A) Heterocyst distribution in the indicated strains grown in bubbled cultures with ammonium and incubated for the indicated times in the absence of combined nitrogen under culture conditions (see legend to Fig. 2 for details). (B) Ratios of the expression levels of *asr2819* in the indicated strains at the indicated times after N step-down, measured by qRT-PCR normalizing with the *rnpB* gene. Data are the mean of two to three independent experiments.

The *Anabaena* ORF *asr2819*, which presumptively encodes an 84-amino-acid protein, was induced upon N step-down (ca. 3- to 4-fold increase after 24 h [Fig. 7B]; see also [30]). To study the role of Asr2819, we sought to generate mutant strains that lack this peptide. Two hundred and forty bp, including all of the *asr2819* ORF but its final 15 bp (see Fig. 1), were removed from the genome of the wild type, the *hetC-p* mutant (CSL16), and *the patS hetN* (CSL11) mutant, generating, respectively, strains CSL97, CSL98, and CSL106. In strain CSL97 the percentage of (pro)heterocysts was somewhat higher, and the mean interval size shorter, than in the wild type (Table 3). Notably, at 72 h this strain showed a very relaxed pattern of (pro)heterocyst spacing, reflected in a greater dispersion of size intervals than in the wild type (Fig. 7A). In strain CSL98, (pro)heterocyst frequency was similar to that of its parental strain (CSL16) (Table 3). Thus, the *hetC-p* mutation is epistatic over the *asr2819* mutation. Like its parental strain (CSL11), strain CSL106 produced a very high proportion of heterocysts (in fact, given the normal stickiness of heterocysts, and thus of filaments, it was problematic to distinguish vegetative cell intervals). Because the *asr2819* gene product contributes to the formation of the spatial pattern of heterocyst distribution along the *Anabaena* filament (Fig. 7A), we name it the *patC* gene.

## Discussion

There has been a long-standing speculation that the HetC protein, which is similar to ABC exporters and required for differentiation, might act by transferring the negative regulators PatS and/or HetN from the heterocysts to the neighboring cells. To address this issue, we have undertaken a study of epistatic effects between the genes *hetC*, *patS* and *hetN*. In a *hetC patS* double mutant (strain CSL1), the frequency of (pro)heterocysts is high, even higher than in the single *patS* mutant after a long incubation in the absence of combined nitrogen. Moreover, strain CSL1 produces heterocyst-specific glycolipids and shows detectable expression of the *nifHDK* genes encoding nitrogenase, although both are found at lower levels than in the wild type. Thus, the lack of the negative factor PatS mitigates the effect of the lack of HetC. In contrast, the lack of *hetN* alone does not alleviate the lack of HetC, although it further increases differentiation in the absence of HetC and PatS. These results would be compatible with the idea of HetC-dependent export of a PatS-derived morphogen. The results would not eliminate the possibility that HetC exports a morphogen that is derived from HetN if one assumes that in the absence of HetC a previous accumulation of PatS in the proheterocyst suffices to inhibit differentiation.

However, two sets of data speak against HetC function being solely PatS (or PatS and HetN) export from the (pro)heterocyst. First, the cells stained with Alcian Blue in the double *patS hetC* (or *patS hetC-p*) and triple *patS hetN hetC* (or *patS hetN hetC-p*) mutants never look like mature heterocysts (see Fig. 3A). Indeed, nitrogenase activity was not effectively complemented, albeit in the double and triple mutants lack of nitrogenase activity could be related to an excessive frequency of differentiating cells, which may result in an unsustainably high ratio of heterocysts to vegetative cells in the filament. Indeed, this appears to be the case already for the *patS hetN* double mutant (strain CSL11), which lacks oxic nitrogenase activity (Table 4). (In this regard, our results with strain CSL11 contrast with the observations, reported in [12], that nitrogenase activity was highly expressed in a *patS* mutant in which *hetN* was diminished in expression.) None of those mutants grew diazotrophically (Table 4). Second, the distribution of heterocysts differs in the *patS hetC* (or *patS hetC-p*) double mutant and in the single *hetC* or *patS* mutants, and differs also between the *patS hetN hetC* (or *patS hetN hetC-p*) triple mutant and the *hetC* or *patS hetN* mutants (Fig. 2, Table 3).

Deletion of the putative peptidase motif of HetC results in a greatly reduced frequency of (pro)heterocysts, albeit not as low as does deletion of *hetC* (Table 3). Because lack of processing of the primary product of *patS* to produce an active peptide results in lack of differentiation [5], the phenotype of strains that bear a *hetC-p* version might be consistent with an involvement of the peptidase domain of HetC in processing of PatS. However, although the HetC-p-GFP fusion protein appears to be correctly localized (Fig. 4C), one cannot be sure that the HetC-p protein retains other possible activities such as any putative transport activity. In a wild-type background, expression of PatS-17 or PatS-8 (a putative product of processing of PatS-17 in the proheterocysts) as the only PatS version recreates a heterocyst pattern similar to that of the wild-type strain, whereas lack of processing of PatS-17 provokes inhibition of differentiation [5]. It might be expected that if the peptidase domain of HetC were involved in processing of PatS-17, expression of PatS-17, but not of PatS-8, in a *hetC-p* background would reduce the frequency of heterocysts. However, both strains CSL101 and CSL102 exhibited low heterocyst frequencies (Table 3), indicating that expression of PatS-8 does not compensate for the lack of the peptidase domain of HetC. In the scenario that HetC were not directly involved in PatS (or PatS and HetN) processing or transfer, the partial compensation of the lack of *hetC* by deletion of *patS* (or *patS* and *hetN*) could be explained by the assumption that the elimination by mutation of the negative factors makes the requirement for HetC, a positive element, less strict during heterocyst differentiation.

To get clues for the function of HetC and HetP, we have studied the subcellular localization of those two proteins in (pro)heterocysts by making use of C-terminal GFP fusion domains expressed from gene constructs present in the cells with copy numbers similar to those of the native genes. In mature heterocysts, HetC-GFP is localized through the heterocyst periphery, and appears especially concentrated near the heterocyst poles. Because different bioinformatics programs predict that HetC has a number of transmembrane segments, it is evident that this protein is targeted to a membrane, and according to Fig. 4C may be targeted to the plasma membrane. In both CSM1 and CSL33, there is more fluorescence near the cell poles. However, it is unclear whether that localization results from the presence of two close membrane units in the heterocyst neck (see e.g. Fig. 2d in [31]), or whether there is a preferential targeting near the cell poles. In Gram-negative bacteria, genes that encode exporters of toxic peptides are frequently linked to genes that encode a protein that belongs to the membrane-fusion protein family that spans the periplasmic space linking the ABC exporter to an outer membrane channel [14]. Because no homolog of membrane-fusion proteins has been detected in the *hetC* genomic region, it has been speculated that the HetC substrate could be released to the *Anabaena* periplasm (see [14]). It is also possible that HetC acts in connection with a different type of protein to promote intercellular molecular transfer. As yet another alternative, HetC might not mediate transport but have a different function that may involve interactions with another membrane factor. Given that HetC has been involved in the inhibition of cell division in the differentiating cells leading to commitment to differentiation [16], HetC might interact with components of the cell-division complex. Such a possibility would be reminiscent of the interaction of the ABC-transporter-like FtsEX complex with FtsZ in *E. coli* [32].

It is notable that inactivation of genes that encode negative factors of heterocyst differentiation, *patS, hetN,* or *patU3* [33], produce a Mch phenotype, which is also the tendency with over-expression of the positive elements *hetP* (Fig. 6) or *hetF* [34], [35]. In contrast, inactivation of *hetC* tends to alleviate the Mch distribution in *patS* or *patS hetN* backgrounds, shifting the heterocyst distribution pattern to a higher frequency of short intervals different from zero (Fig. 2, Table 3). The tendency to differentiation in clusters of cells when the negative regulators are eliminated could involve the action of a putative PatN factor if its role in *Anabaena* were similar to that described in *Nostoc punctiforme* at establishing clusters of cells permissive for differentiation (those lacking PatN) by biased inheritance during cell division [36]. PatN is a membrane factor and its inactivation results in an Msh (increased frequency of single heterocyst) phenotype. It is tempting to speculate that the roles of HetC and PatN could be related, e.g., HetC might act only in cells devoid of PatN.

HetP-GFP is present throghout the cell area in proheterocysts, and later is almost restricted to the polar regions, where it remains in mature heterocysts (Fig. 5C). HetP-GFP proteins appear to be present in soluble fractions from whole *Anabaena* filaments, which could correspond to the protein present throughout proheterocysts, and in membrane fractions, which could correspond to the protein in cells more advanced in differentiation, 12 h after N step-down (our unpublished observations). Several bioinformatics programs (Psipred, TMHMM) predict that HetP bears a single putative membrane segment comprising residues ca. 29-44 (Fig. 5B). This segment could maintain HetP anchored to membranes at the cell poles. Moreover, the HetP-GFP fluorescence near the heterocyst poles appears peripheral to the chlorophyll fluorescence in those cells. It would be of much interest to study the mechanism that sorts this protein to membranes specifically at the heterocyst poles. As a possibility, membrane regions with a specific curvature could be selected in the differentiated and complex heterocyst pole, as is the case for the localization of SpoVM during spore formation in *Bacillus subtilis* [37]. Also, HetP could be anchored to the membrane and maintained in the heterocyst poles by interaction with septal proteins. Our results on epistasis of *hetC* and *hetP* show that a moderate over-expression of *hetP* (in strain CSL68) fully complements the lack of a functional *hetC* gene (*hetC-p*; Fig. 6). It is worth to stress that in strain CSL68 nitrogenase activity and diazotrophic growth are similar to those in the wild type (Table 4), in contrast to a partial complementation of the *hetC* mutation by PatS deletion (in strain CSL1) or of *hetR* inactivation by *hetP* over-expression [18]. These results are consistent with a functional link between HetC and HetP, whose roles may overlap. This idea is also supported by the similar effects of the mutation of each gene that results in strains capable of initiation of differentiation, which however arrests at an early stage.

In summary, our results are consistent with a double role of HetC at regulation of heterocyst differentiation. HetC might be involved in processing or transfer of the negative regulators (PatS and HetN) from (pro)heterocysts to the neighboring cells. Besides that, HetC (and HetP) might interact with membrane factors leading to inhibition of cell division, perhaps in cells devoid of PatN. In this model, PatN might not influence the selection of cells that initiate differentiation but those in which, though the action of HetC, differentiation could proceed to completion. On the other hand PatC, which is not necessary for differentiation, might participate in differentiation site selection especially during established diazotrophic growth. Finally, the commitment to differentiation could involve still more elements. It is noteworthy that the inactivation of *sepJ*, which encodes a septal protein that is important for cell-to-cell anchoring and intercellular transfer in the filament, interferes, as is the case of *hetC*, with inhibition of cell division during heterocyst differentiation [24]. Deciphering the structure of the multiprotein complexes at the vegetative cell-heterocyst septa represents an outstanding research challenge for the future.
